# An Epidemic of Kine-Pox

**Published:** 1871-05

**Authors:** 


AN EPIDEMIC OF KINE-POX—THE AGE OF JENNER RETURNING.—Quite a sensation has been produced very recently in professional circles in SanFrancisco, by the discovery that an epidemic of kine-pox has broken out among the cattle on the dairy ranches of Marin County, the disease extending to the hands of milkers, as in the days of Jenner. A portion of the virus in the form of crusts has been procured and used for vaccination, producing what is beyond a doubt the true vaccine disease. Several of our physicians, especially Drs. McMillan and Trask, have conducted these experiments. Perhaps it is too soon to make a full report on the subject. In another month, however, we shall be prepared, from personal observation, and from the testimony of the gentlemen above named and other experimenters, to give our readers a complete account of the outbreak of the disease among the cattle, and its character when transferred to the human system. Although we are not among those who believe that vaccinia has lost anything of its virtue by transmission in the human subject, yet it would be highly satisfactory to have a renewal of the supply of virus from a spontaneous development of the disease in the rare form of an epidemic. —*Pacific Medical Journal.*

